# MEMS Smart Glass with Larger Angular Tuning Range and 2D Actuation †

**DOI:** 10.3390/mi16010056

**Published:** 2024-12-31

**Authors:** Md Kamrul Hasan, Mustaqim Siddi Que Iskhandar, Steffen Liebermann, Shilby Baby, Jiahao Chen, Muhammad Hasnain Qasim, Dennis Löber, Roland Donatiello, Guilin Xu, Hartmut Hillmer

**Affiliations:** 1 Institute of Nanostructure Technologies and Analytics (INA), Technological Electronics Department and Center for Interdisciplinary Nanostructure Science and Technology (CINSaT), University of Kassel, Heinrich-Plett-Straße 40, 34132 Kassel, Germany; chen@ina.uni-kassel.de (J.C.); qasim@ina.uni-kassel.de (M.H.Q.); d.loeber@ina.uni-kassel.de (D.L.); donatiello@ina.uni-kassel.de (R.D.); hillmer@ina.uni-kassel.de (H.H.); 2 Nanoscale Glasstec GmbH, Heinrich-Plett-Straße 40, 34132 Kassel, Germany; mustaqim.iskhandar@nanoscale-glasstec.com (M.S.Q.I.); steffen.liebermann@nanoscale-glasstec.com (S.L.); shilby.baby@nanoscale-glasstec.com (S.B.); guilin.xu@nanoscale-glasstec.com (G.X.)

**Keywords:** optical MEMS, smart glass, electrostatic actuation, pull-in, daylight steering, 2D actuation, stress concentration

## Abstract

Millions of electrostatically actuatable micromirror arrays have been arranged in between windowpanes in inert gas environments, enabling active daylighting in buildings for illumination and climatization. MEMS smart windows can reduce energy consumption significantly. However, to allow personalized light steering for arbitrary user positions with high flexibility, two main limitations must be overcome: first, limited tuning angle spans by MEMS pull-in effects; and second, the lack of a second orthogonal tuning angle, which is highly required. Firstly, design improvements of electrostatically actuatable micromirror arrays are reported by utilizing tailored bottom electrode structures for enlarging the tilt angle (Φ). Considerably larger tuning ranges are presented, significantly improving daylight steering into buildings. Secondly, 2D actuation means free movement of micromirrors via two angles—tilt (Φ) and torsion angle (θ)—while applying two corresponding voltages between the metallic micromirrors and corresponding FTO (fluorine-doped tin oxide) counters bottom electrode pads. In addition, a solution for a notorious problem in MEMS actuation is presented. Micromirror design modifications are necessary to eliminate possible crack formation on metallic structure due to stress concentration during the free movement of 2D actuatable micromirror arrays. The concept, design of micromirror arrays and bottom electrodes, as well as technological fabrication and experimental results are presented and discussed.

## 1. Introduction

The smart window concept [1,2,3,4,5,6,7] in dynamic daylight control application for energy-saving purposes in lighting, heating and cooling to counter CO_2_ emissions and global warming [8,9,10] has been a continuous research field for decades. Smart switchable glasses allow the dynamic control of sunlight access into buildings for better human health and well-being [11,12,13]. There are many commercially available smart glass technologies for daylighting such as SPD, PDLC, electrochromic, thermochromic switchable glasses, etc., [2,3,14,15,16,17,18] to fulfill the requirements of an optimum daylighting system [19,20]. Along with the many advantages of these technologies, drawbacks like speed, cost, tint, blockage, temperature dependency and stability must also be considered [19,21,22].

**Figure 1:**
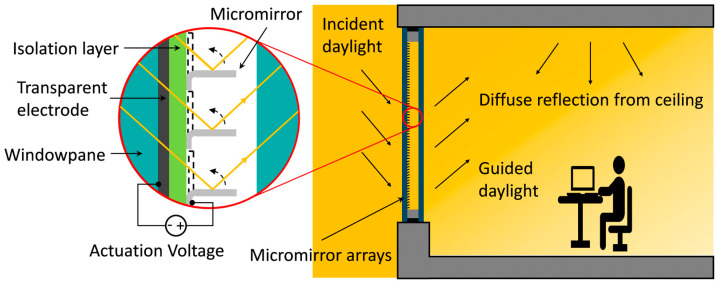
Schematic illustration of MEMS smart window concept where electrostatically actuatable micromirror arrays are arranged in between windowpanes in inert gas environment, allowing active daylight steering inside the building without glare effect. Modified figure from [28].

Optical MEMS-based smart window concepts [23,24,25,26,27], incorporating electrostatically actuatable micromirror arrays arranged in insulation glazing in an inert gas atmosphere, allow active daylight steering into buildings for lighting, heating and cooling. The system considers season, window orientation and user movement inside the room without disturbing the user with glare effects (Figure 1). Mechanically stable micromirrors are invisible to the naked eye from a distance of more than 25 cm, exhibiting long lifetimes, significant energy-saving potential, high cost-effectiveness, fast switching speed, a wide operating temperature range, resistance against solar UV radiation, low power consumption and high resistance against harsh environments (see our reliability study) [23].

**Figure 2:**
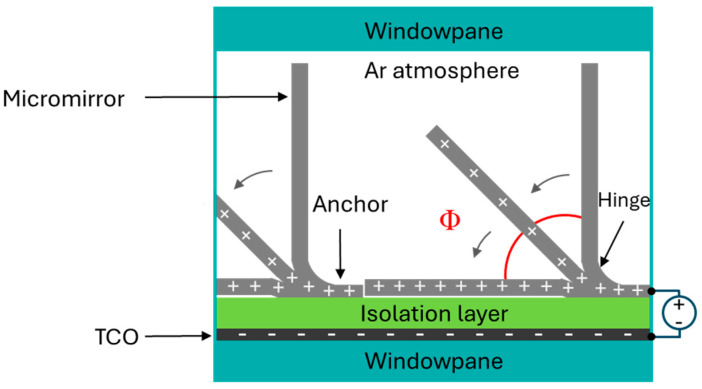
Schematic demonstration of micromirror working principle arranged inside windowpane in inert gas environment, revealing all the major components.

Individual micromirrors consist of a fixed anchor, a properly bent hinge due to tailored stress in metal thin layers and a planar mirror plane via localized stress compensation. The mirror is normally in an open state (90° with respect to the substrate) with no voltage applied (Figure 2). When a potential difference is applied between the mirror (top electrode) and transparent bottom electrode (FTO), both insulated from each other, the micromirrors start to move towards the substrate due to electrostatic attraction forces. Micromirrors can be held in several intermediate tilt angles (Φ) by balancing the electrostatic attraction (due to applied voltage) and the elastic restoring force (bending of hinges) before reaching the critical pull-in point, after which the micromirrors close abruptly on the substrate—a well-known phenomenon in MEMS [29,30,31,32]. The initial tilt angle Φ at 0 V is defined by the residual stress at the hinge, and the tilt angle range ΔΦ is limited by the pull-in effect.

**Figure 3:**
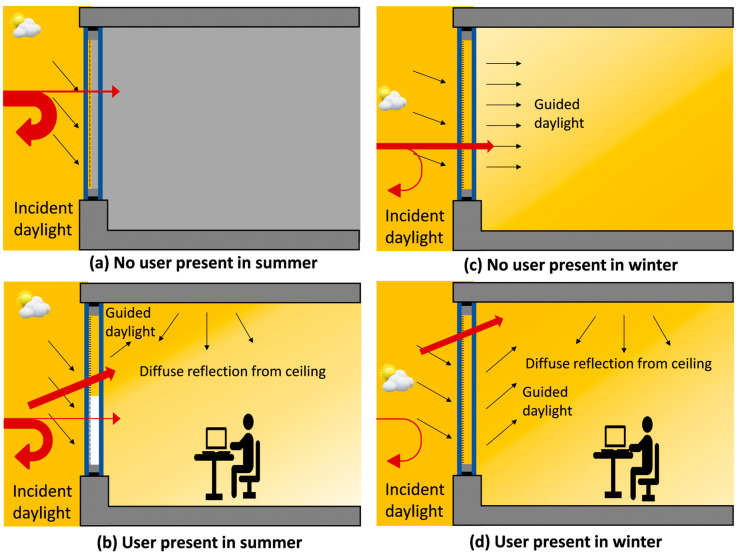
Schematics illustration of MEMS smart windows for daylight steering inside buildings in four different scenarios with and without user presence. (**a**) Summer without user: most of the solar radiation is blocked and reflected outside; thus, the room remains cool. (**b**) Summer with user present: micromirror only at the upper part of the window is kept open to allow daylight entering the room, and most of the room areas remain shaded and cool to reduce cooling load. (**c**) Winter without user: most of the solar radiation is desired to enter inside, heating the walls due to infrared radiation, which acts later as radiation heater. (**d**) Winter with user present: most of the sunlight is guided towards the ceiling without glare effect; thus, the ceiling acts as radiation heater, which reduces the heating load significantly. Black arrows represent the visible daylight guiding path, and red arrows symbolize the heat radiation entering or reflecting to outside, respectively. See Supplementary Materials.

The data from the intelligent sensing system, which monitors the brightest spot of the sky, user movement inside the room and temperature inside and outside, are fed to the control unit to execute the functionality of the micromirror arrays according to the requirement. Depending on all these parameters, a large number of diverse situations are possible; four such scenarios are presented in Figure 3. Energy-saving calculations were executed using the self-developed universal simulation tool “*SAVINGS*” for MEMS smart window in comparison to conventional window blinds, which can be used in any place in the world where a meteorological dataset is available. In the calculation of energy saving using our simulation tool “*SAVINGS*”, we considered a model office room (plain office) in Kassel. We used real weather data, acquired from a long observation period (10 years) for the calculation. The weather data consist of temperature, cloud coverage, irradiance, wind velocity and others as a function of daytime and date over the whole year. Furthermore, future predicted weather data were also used in our calculations for higher accuracy. The energy calculation was further extended for four different cities in Germany (Flensburg, Kassel, Winterberg and Konstanz), as well as two cities outside Germany (Qingdao in China and Riyadh in Saudi Arabia). These results are published in Ref. [25].

In addition to plain office simulations, we calculated the energy balance for a residential apartment, considering typical daily activities in all details of the occupants. The latter one also considered temperature-dependent leakage currents in MEMS smart glass. These calculations reveal that MEMS smart windows can save up to 35% of energy and 30% of CO_2_ emissions.

Currently, the actuation mechanism allows micromirror movement from a free-standing vertical open position (90°) to the closed position (0°) parallel to the substrate. When the voltage is removed, the micromirror returns to the vertical position (90°). This open–close movement of the micromirror is associated with the tilt angle Φ, referred to as 1D actuation. In this case, the usable angle range ΔΦ is between Φ_2_ = 90° and the pull-in angle Φ_pi_, and is only sufficient for daylight guiding in some cases. To serve all cases, it is of advantage to include the backbend tilt angles Φ_3_, which are > 90° [33,34]. However, having smaller pull-in angles Φ_pi_ might also be desirable to obtain higher flexibility in micromirror actuation angles. To achieve smaller pull-in angles Φ_pi_., design improvements of bottom electrodes are necessary to partially overcome the pull-in effect, which is crucial for the improvement in application of micromirror arrays in smart windows.

Moreover, efficient daylight guiding towards the building is limited to certain sun positions with only the open–close movement of the micromirror. Therefore, an additional function of the micromirror, sideways deflection—referred to as 2D actuation—is required to allow flexible daylighting at more possible sun positions throughout the day. Further explanation about 1D and 2D actuation will be added in the forthcoming section.

In this paper, the design improvement of a transparent bottom electrode structure in a 1D actuated micromirror for large angular tuning—via lowering smaller pull-in angles Φ_pi_—is presented in Section 2. Subsequently, the design optimization and implementation of 2D actuation in micromirror arrays for efficient daylight steering are addressed in Section 3.

Additionally, curled microshutter arrays can also be implemented in smart windows. However, curled microshutter arrays allow light modulation only, no light steering. For curled microshutter smart window applications, the literature reveals electrostatic actuation [7,35,36] and electrothermal actuation [37]. In general, electrothermal actuation can be achieved by very small actuation voltages, but high currents are required, and electrostatic actuation needs much higher voltages but operates with very low currents. Power consumption as the product of both is interesting: for our electrostatically actuated micromirrors, the power consumption is very low (0.0018 µW/cm^2^ for 1.2 μm SiO_2_ at 20 °C, 40 V). For the electrothermal actuation of microshutters, much higher power consumption has been reported (910 mW/cm^2^ at 3 V).

## 2. Large Angular Tuning Range for 1D Actuation via Smaller Pull-In Angles, Φ_pi_

**Figure 4:**
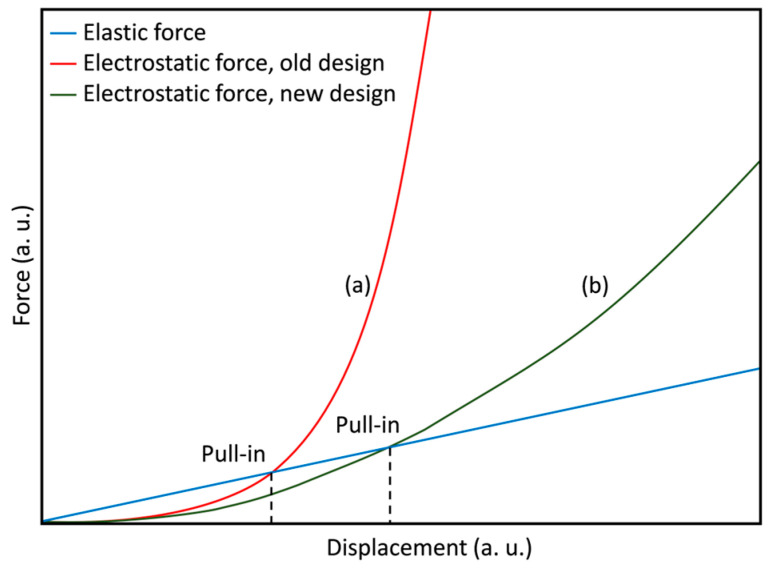
Schematic plots of the electrostatic attraction force as a function of displacement depending on the electrode formation: (**a**) parallel plate configuration and (**b**) triangular shaped bottom electrode (FTO). Pull-in point can be shifted with electrode formation. The elastic force is simplified and shown here in ideal linear relation. The axes are given in arbitrary units (a. u.), as the graphs intend to present the quantitative nature rather than precise quantitative values.

Our micromirrors, with typical dimensions of 400 µm × 150 µm, are normally in open state (~90°) with no applied voltage. By applying a voltage between the micromirrors and bottom electrode (FTO), the micromirrors start to move towards the substrate due to electrostatic attraction force. Micromirror arrays can be held in several intermediate tilt angles before reaching the critical pull-in point by balancing the acting forces (electrostatic and elastic restoring force), after which the micromirrors close abruptly, falling parallel to the substrate. It is well known from the parallel plate capacitor that the electrostatic force is already strongly nonlinear (Figure 4). Thus, micromirror arrays experience abrupt closing when the repulsive elastic force is unable to balance the attractive force.

**Figure 5:**
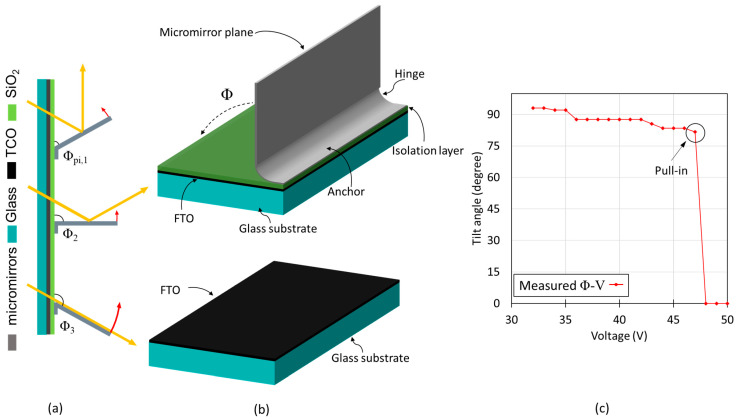
Schematic illustration of different opening angles of micromirrors, (**a**) close to pull-in (Φ_pi,1_), 90° (Φ_2_) and back bending angle (Φ_3_). (**b**) Micromirror configuration with isolation layer and unstructured FTO. (**c**) Corresponding tilt angle Φ versus voltage characteristics of micromirror array with unstructured FTO.

The abrupt closing of the micromirror arrays beyond the pull-in point results in a smaller range of intermediate tilt angles, which limits the daylight steering functionality of MEMS smart window into the building. The pull-in effect of micromirror with unstructured FTO can be observed in the tilt angle–voltage curve, as well as via capacitance measurements as a function of voltage [33,34,38]. Starting from 0 V, the tilt angle Φ decreases continuously with increasing applied voltage, resulting in a continuous increase in capacitance towards its maximum value when all mirrors are closed. It can be observed in Figure 5c that only a small tilt angle range can be achieved by the micromirror before reaching the pull-in point. This limits the flexibility of daylight steering functionality of micromirror arrays in smart windows.

**Figure 6:**
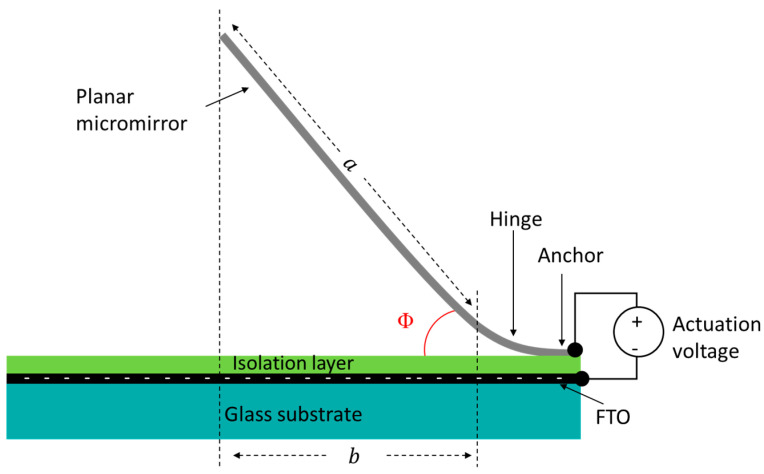
Schematic illustration of the cross section of a single micromirror and the part of the substrate (indicated by the distance *b*) covered by the planarized part of the micromirror (length *a*), which are used to calculate actuation tilt angle *Φ* calculation during the electrostatic actuation.

Since the precise actuation angle measurement for a single micromirror out of such large micromirror arrays during the actuation is yet to be established, the actuation angle measurement is carried out via the following method, as illustrated in Figure 6. The distance of the planar micromirror plane from curled hinge *a* and the distance of the substrate covered by the micromirror *b* are measured using high-resolution optical microscope at each voltage step during the electrostatic actuation, followed by the calculation of tilt angle using the inverse cosine function. The distance of planar micromirror plane *a* is identified during the fabrication process, which defines the actuation tilt angle Φ, i.e., the angular displacement of a planar micromirror.

### 2.1. Concept and Design

**Figure 7:**
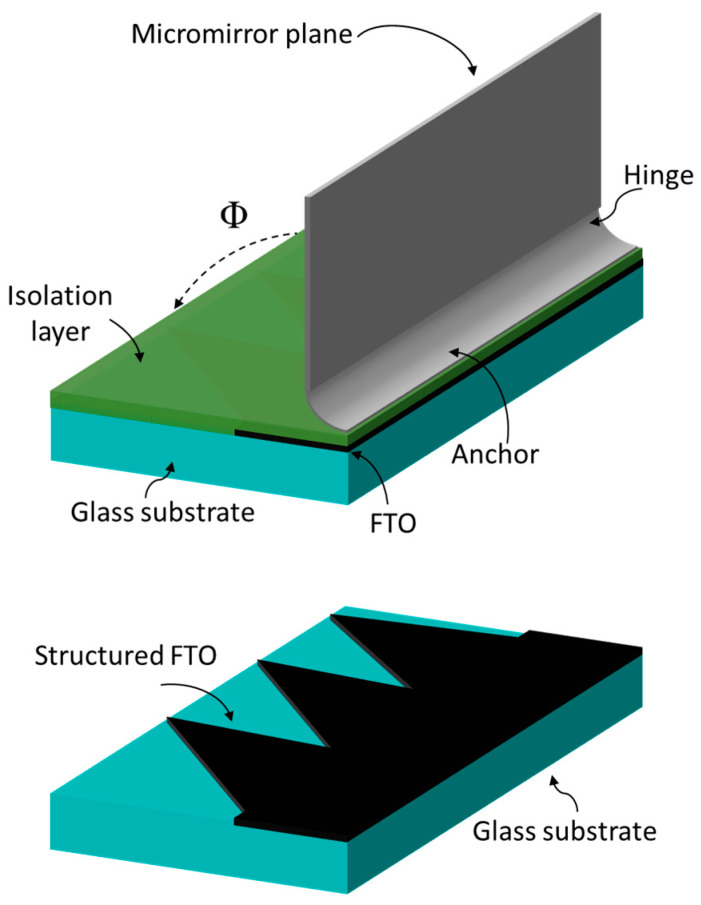
Schematic representation of micromirror configuration with isolation layer and laterally structured FTO.

The abrupt closing of a micromirror beyond the pull-in point results from the strong nonlinear increase in the electrostatic force when micromirror planes come closer to the bottom electrode. The gradual reduction in the overlapping electrode area can reduce the electrostatic attraction force to a smaller finite value (Figure 4) beyond the pull-in point as the micromirror approaches the bottom electrode (FTO). The bottom electrode is structured in a triangular shape to allow a gradual reduction in the electrode overlapping area, allowing a larger angle range ΔΦ and, thus, more possibilities of intermediate tilt angles during actuation (Figure 7).

### 2.2. Technological Fabrication Process

**Figure 8:**
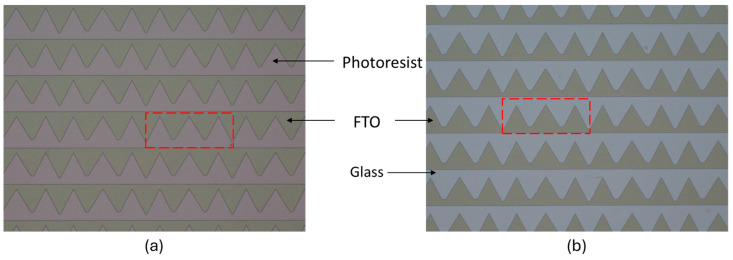
Micrograph of FTO structure after (**a**) photolithography with photoresist and (**b**) wet chemical etching process and photoresist removal. Area of the FTO structure corresponding to a single micromirror is visualized by the red dashed lines.

The technological fabrication process of micromirror arrays begins with the FTO structuring process using wet chemical etching based on the FTO etching method invented by McLean et al. [39]. The wet chemical etching process uses zinc (Zn) particles and hydrochloric acid (HCl) for metal–acid etching and FeCl_3_ as an oxidation and penetration control agent, which prevents under-etching. Photoresist structuring is executed in a photolithography process, which acts as etch mask. Zinc particle size has a significant influence on the efficiency of the ETO etching process. The substrate with a photoresist etch mask is submerged into the etching solution containing HCl (1.27 M) and FeCl_3_ (0.02 M) for approximately 60 s. Tin oxide (SnO_2_) is reduced to Sn (tin) by redox reaction, whereas excessive removal of FTO is prevented by the formation of an iron layer from the reaction between Zn and penetration control agent (Fe^2+^), which is produced via the reaction of Sn with the metal dissolution agent (Fe^3+^) from FeCl_3_. Deionized (DI) water is used to stop the wet chemical etching process, followed by immersion into the cleaning solution made of HCl (1.26 M) and FeCl_3_ (0.03 M) for 5 min to remove the remaining Sn and the reduced penetration control metals. The photoresist etch mask is removed using stripper solution DMSO (dimethyl sulfoxide) at 80 °C for 5 min. Detailed investigation of different photolithography and wet chemical etching process parameters was executed to identify the optimum process parameters (Figure 8).

**Figure 9:**
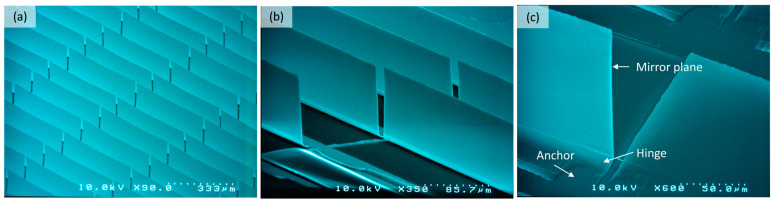
SEM micrograph of (**a**) planarized free standing micromirror arrays, (**b**) a magnified area, and (**c**) revealing major components of micromirror, fixed anchor, bend hinge and planar micromirror plane.

An isolation layer (SiO_2_) with a thickness of approximately several 100 nm is deposited on FTO structures using a PECVD process. For the fabrication of micromirror arrays on glass substrate with FTO and SiO_2_, a UV lithography process is executed to define the micromirror structure on the sacrificial photoresist layer. A special bi-layered photomask [40] was used, capable of forming leading edge and undercut in a single exposure step. A metal layer stack of multiple metal thin layers combining Al and Cr is deposited via EBPVD (electron beam physical vapor deposition) with thickness ranging within 60–100 nm and 10–40 nm of Cr, respectively. It is important to note that the thicknesses are given in ranges since they vary by design, desired opening angle, and, as necessary, finetuning to counter processing fluctuations. Subsequently, a lift-off process at 80 °C is executed to remove the sacrificial photoresist layer. Thereafter, the sample is dried using Marangoni drying or IPA vapor drying process to achieve free standing planar micromirror arrays (Figure 9).

### 2.3. Experimental Characterization of Micromirrors with 1D Actuation

**Figure 10:**
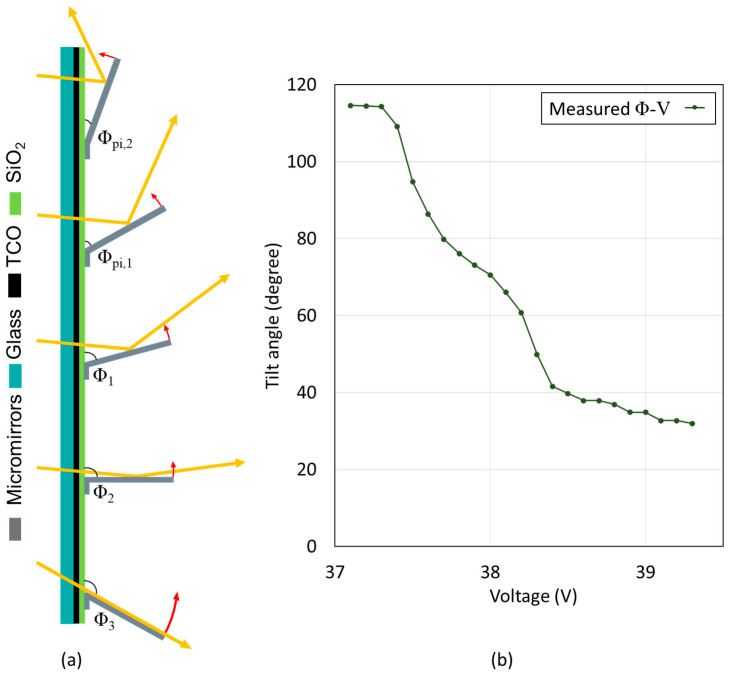
(**a**) Schematic illustration of different actuation tilt angles of micromirrors achieved by structured bottom electrode (FTO): new pull-in (Φ_pi,2_) with structured FTO, previous pull-in (Φ_pi,1_) with unstructured FTO, an intermediate tilt angle (Φ_1_), 90° (Φ_2_) and backbend angle (Φ_3_). A situation of shallow incoming angles is selected, which is the most challenging case. (**b**) Corresponding tilt angle Φ versus voltage characteristics of micromirror array with unstructured FTO.

After successful fabrication, the micromirror array module is mounted in an inert gas environment to keep humidity and O_2_ away to eliminate the possibility of negative impacts on the micromirror actuation mechanism. The electrostatic actuation process is observed under a microscope. The angle measurement and calculation are performed in the same procedure explained in Figure 6. Experimental results show that the micromirrors can be held within a larger tilt angle span ΔΦ. Since the pull-in angle is shifted to smaller values (Figure 10), this allows for efficient daylighting with more flexibility in tilt angles in comparison to the micromirror arrays with unstructured FTO.

Daylight steering during both high and low sun positions can be achieved by combining the structured FTO with back-bended micromirror, i.e., with tilt angles Φ_3_ of more than 90° in default open state. It is important to note that our optical MEMS smart glass is a platform technology, which has different alternative applications, where the wide flexibility of tilt angle creation will also be highly valuable.

The inclination of tilt angle achieved was determined by the DC voltage applied between the top and bottom electrodes. This relation is not linear and is only defined until the pull-in effect occurs, i.e., where the micromirrors close completely. While voltages of a few volts can be applied at low tilt angles, a more precise setting of the actuation voltage in the millivolt range with an accuracy of hundredths or tenths of a volt is required until the defined pull-in threshold value is reached. Since the relationship between voltage and tilt angle is reproducible, a control circuit can perform the steering precisely having this relation as a database.

## 3. Implementation of 2D Actuation in Micromirror Arrays

### 3.1. Concept of 2D Actuation

**Figure 11:**
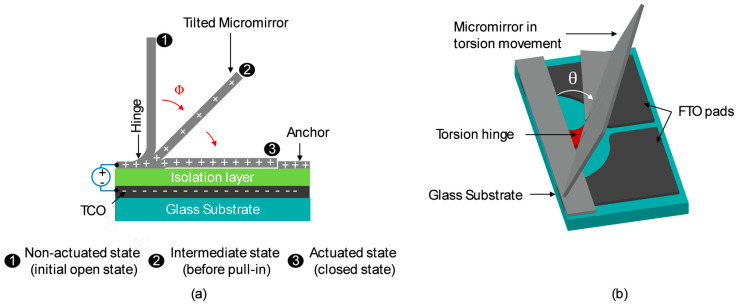
Schematic illustration of micromirror generating (**a**) tilt angle Φ and (**b**) torsion angle θ due to electrostatic attraction force.

The MEMS smart window concept utilizes the movements of micromirror arrays for personalized light guiding inside the building via the deflection of sunlight towards the specific part of the ceiling area in the room depending on the position of the user. When a micromirror can only create tilt angles Φ in actuation, it is defined as 1D actuation. As presented in the previous section, micromirror actuation with structured FTO allows higher accuracy and flexibility in daylighting due to larger tilt angle ranges ΔΦ. There is another possibility of creating torsion angle θ in actuation by integrating a flexible torsion spring-like hinge in micromirror design and specific structured FTO by adjusting the electrostatic force between the mirror and the counter bottom electrodes. When the tilt angle Φ and torsion angle θ can be combined in the micromirror actuation, then it is defined as 2D actuation (Figure 11).

**Figure 12:**
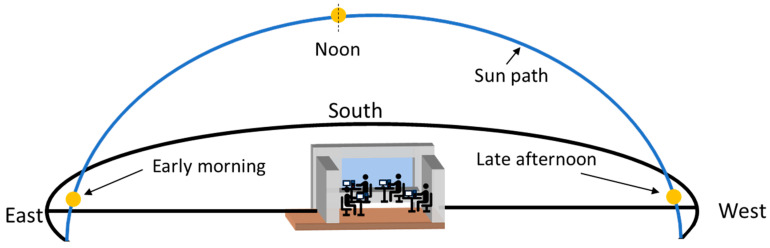
Schematic presentation of a model room equipped with south-oriented MEMS smart window, illustrating sun path and different sun positions throughout the day. Modified figure from [27] with permission of Leuze Publishing House.

Considering a south-oriented window (Figure 12), 1D actuation is highly efficient at noon time for daylight guiding when the sun has high altitude angle. However, 1D actuation has limited functionality in light steering in the low sun position, specifically in the early morning and late afternoon, as well as during the sideways movement of the user. Hence, personalized active light guiding can be interrupted due to different sun positions in the sky throughout the day, thus potentially limiting the envisioned application of micromirror arrays in the actual window. Therefore, the implementation of 2D actuation in micromirror arrays is required to ensure active light guiding throughout the day, independent of the sun position in the sky and user movement in the room.

**Figure 13:**
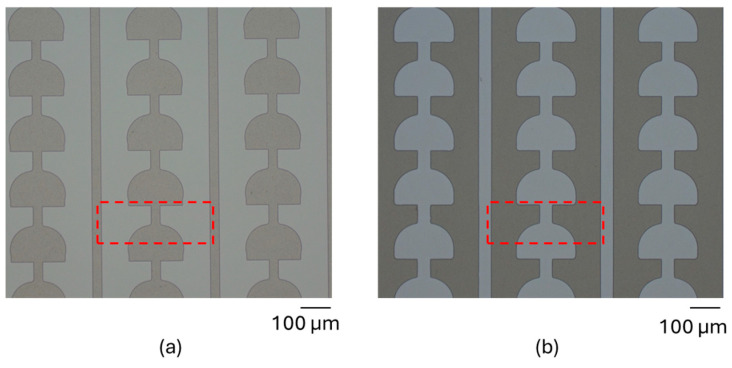
Micrograph of FTO structure after (**a**) photolithography with photoresist and (**b**) wet chemical etching, cleaning process and photoresist removal. Corresponding area of FTO structure for a single micromirror is defined by the red dashed lines. Original figure from [27] with permission of Leuze Publishing House.

**Figure 14:**
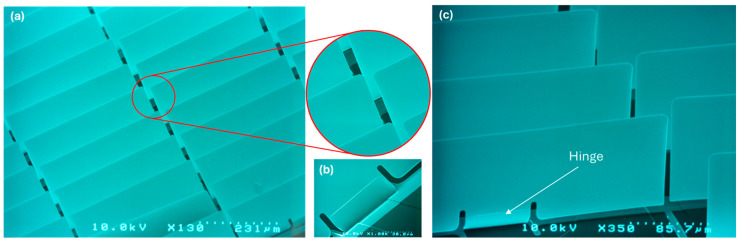
SEM micrograph of (**a**) free standing planar micromirror arrays after lift-off and drying process with an inset of magnified area, (**b**) torsion spring hinge and (**c**) hinge area at a different opening angle to distinguish the vertical opening state of the micromirror, revealing structured FTO pads as well as narrow gap between torsion spring hinge and micromirror plane. Original figure from [27] with permission of Leuze Publishing House.

As elaborated previously in Section 2.2, the fabrication of 2D actuatable micromirror arrays starts with the FTO structuring process using wet chemical etching process (Figure 13), followed by the deposition of isolation layer on top, and afterward, the fabrication of planar micromirror arrays (Figure 14) using photolithography, metal deposition, wet etching, lift-off and drying processes.

**Figure 15:**
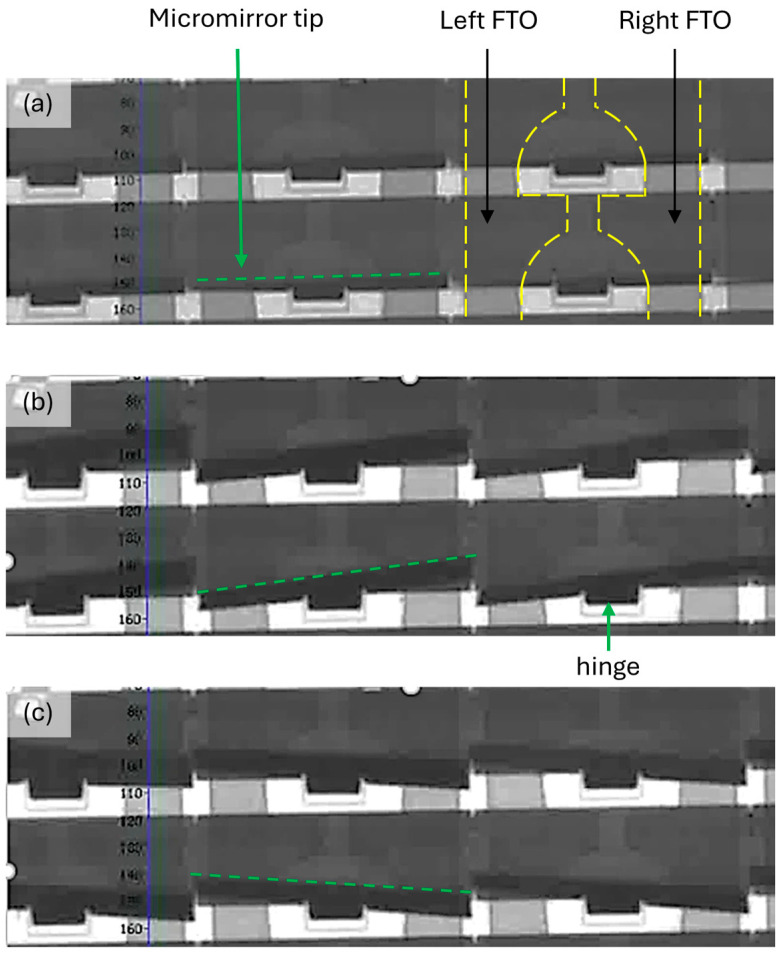
Micrographs of micromirror arrays in (**a**) free-standing open position, and (**b**) when micromirrors are actuated towards the right and (**c**) left while applying voltage between micromirror and FTO pads. The micrographs are taken in reflection mode and the angle is shown with a green dashed line. Modified figure from [27] with permission of Leuze Publishing House.

Efficient etching of FTO results in the electrical resistance of higher than 20 MΩ between FTO pads which is sufficient to isolate them. Thus, the voltage can be applied on the FTO pads and micromirror arrays independently without crosstalk. The micromirror array module is housed in a chamber in an inert atmosphere, sealed with butyl for further electrostatic actuation. The complete actuation setup is placed under a transmission microscope to observe and record the micromirror movement during the electrostatic actuation process. A multichannel waveform generator is used to bias the micromirrors (top electrode) and counter FTO pads (bottom electrode) independently. When voltage is applied between micromirror and the right FTO pad, the micromirror generates a torsion angle towards right, whereas a leftwards torsion angle is achieved by applying voltage between the micromirror and left FTO pads. A torsion angle θ can be varied from 5° to 12° in right and left directions by varying the voltage from 70 V to 130 V, which changes the tilt angle Φ as well. One such actuated situation is presented in Figure 15, where the light and dark grey parts (FTO pads) are located on the substrate plane, and the black parts show the free-standing micromirror. The dark grey represents the transmissive regions. The torsion angles θ in Figure 15 are highlighted by green dotted lines. The tilt movement of a micromirror is implemented via a voltage applied on the micromirror and both FTO pads (on same potential). When the potential difference is removed, the micromirrors return back to their original positions without stiction to the substrate.

### 3.2. Elimination of Cracks in Metallic Micromirror Due to Induced Stress Concentration

**Figure 16:**
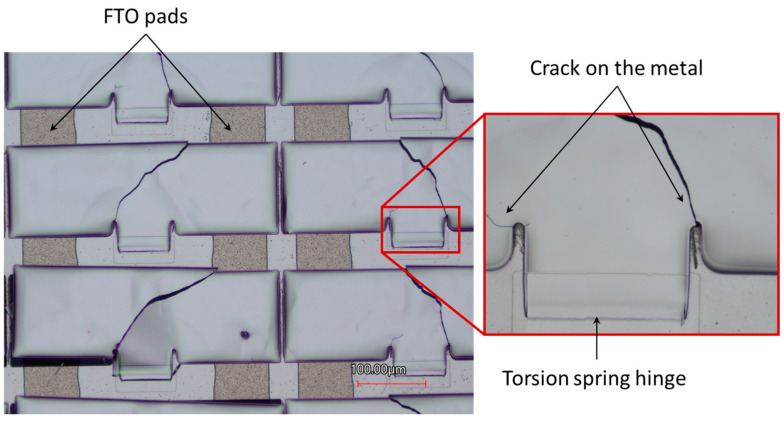
Micrograph of crack formations at the narrow gaps between torsion spring hinge and micromirror plane during actuating micromirror arrays with an inset of higher magnification.

During the movement of micromirrors via electrostatic attraction force, crack formation in metallic micromirrors is observed in many cases at the edge of the narrow gap (<5 µm) between the torsion spring hinge and micromirror plane (Figure 16). This results from the induced stress concentration—a common phenomenon in mechanical structures that leads to the complete damage of the micromirrors. The unwanted deformation leads to a compromise of reliability and overall performance, which limits the potential application of micromirror arrays in smart windows.

**Figure 17:**
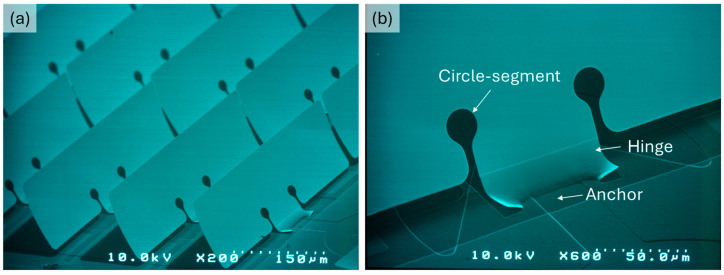
SEM micrograph of micromirror arrays with new design integrating a circle-segment. (**a**) Planarized free standing micromirror arrays and (**b**) magnified hinge area, revealing major components of micromirror, fixed anchor, bend hinge and planar micromirror plane.

Therefore, micromirror design modifications are required to eliminate the crack formation on the metallic structure by reducing the stress concentration at the joint of torsion spring and micromirror plane. From the literature on stable mechanical structures [41,42], stress concentration can be reduced by adding a rounding shaped structure at the gap end between the micromirror and torsion spring. Therefore, the micromirror design is modified by integrating a defense hole system—a circle-segment at the end of the slit. The torsion spring hinge and micromirror plane allow stress distribution during the free movement of the micromirror during electrostatic actuation (Figure 17).

### 3.3. Experimental Characterization of 2D Actuatable Micromirrors

**Figure 18:**
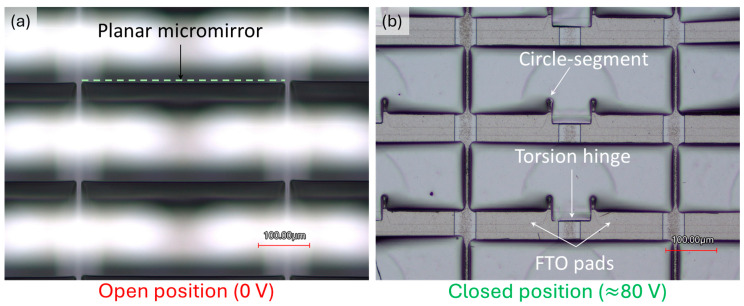
Micrograph of planarized micromirror arrays with new design integrating circle-segment at (**a**) open position at 0 V and (**b**) closed position at approximately 80 V without any crack formation due to stress distribution.

As mentioned in the previous section, the micromirror array module is housed in an inert gas environment after fabrication to eliminate unwanted external effects during electrostatic actuation. The left and right FTO pads, as well as the micromirror arrays, have separate contact areas of the substrate. A multichannel waveform generator is used to apply voltages to the micromirror arrays and FTO pads independently. The movement of micromirror arrays in electrostatic actuation is observed under a microscope. In Figure 18, the micromirror array movement from open to closed states during electrostatic actuation is observed without any damage due to stress-concentrated crack formation. The micromirror arrays return back to their original open state when the actuation voltage is removed.

MEMS micromirror arrays are fabricated without including an anti-stiction layer. Many open/closed/open cycles were performed. Luckily, stiction was no problem. The characterization of our MEMS micromirror arrays was performed by recording C-V curves and other methods to identify the actuation voltage, temperature range, speed, leakage current and other important characteristic parameters of the micromirror arrays. Moderate threshold voltages where the micromirrors reach pull-in point were identified. Therefore, higher voltages that might result in permanent stiction can be avoided.

## 4. Conclusions

A small range of actuation tilt angles can severely limit the functionality of MEMS smart window. An innovative method to achieve larger actuation tilt angle spans for micromirror arrays in smart window by partly overcoming pull-in limitations is presented. The transparent counter bottom electrode (FTO) is structured in a specific way to reduce the electrostatic attraction force to a finite value when the micromirror moves closer to the FTO surface. Thus, the increase (extreme nonlinearities in the Φ = Φ(V) curve) in the electrostatic attraction force near the pull-in point is avoided during the actuation with tailored FTO structuring. The pull-in point is shifted to a higher voltage and smaller tilt angle. This further allows efficient daylight steering with precise control and a large angle tuning range. Moreover, the implementation of 2D actuation (tilt angle Φ and torsion angle θ) by integrating a torsion spring hinge and structured FTO is presented. A torsion angle range of 5–12° for the applied voltage from 70 to 120 V is measured. Efficient daylight guiding throughout the day independent of sun position and user movement can be achieved with 2D actuatable micromirror arrays. Additionally, the torsion spring hinge is modified, incorporating a circle-segment to eliminate crack formation due to stress concentration during the free movement of the micromirror. The unwanted deformation of micromirrors during electrostatic actuation is avoided by redistributing the concentrated stress. Our micromirror arrays are a platform technology that is intended to be implemented in many other applications where switching speed is significantly important. A typical switching speed in the range of 20 µs, with a record value of 1 µs, was measured.

## Data Availability

The original contributions presented in this study are included in the article. Further inquiries can be directed to the corresponding author.

## Supplementary Materials

The following supporting information can be downloaded at: https://www.mdpi.com/article/10.3390/mi16010056/s1, Figure S1: Illustration of solar blackbody spectrum (black curve), solar radiation at sea level (blue curve) and blackbody radiation (red curve) at 24 °C inside a room (scaled-up by 55 times for better visibility) [43]; Figure S2: Schematic demonstration of the solar infrared absorption by the wall inside a room in a cold winter day without any user present. The intensity of red color symbolizes the amount of absorbed and re-emitted heat energy. The visible and near infrared radiation (solar infrared) is absorbed inside the room.
