# Supplementary material for: MEMS Smart Glass with Larger Angular Tuning Range and 2D Actuation
Source: Micromachines (Basel). 2024 Dec 31;16(1):56. doi: 10.3390/mi16010056 (PMC11767687; doi:10.3390/mi16010056)
Supplement: Supplementary file 1 [file micromachines-16-00056-s001.zip › micromachines-3372582-supplementary.pdf]

Supplementary Materials

The solar spectrum is shown in Figure S1, which reveals the black body radiation of the sun (at a temperature of 5250 °C proving a peak at $\lambda = 500$ nm) by the full black line. The irradiance of this spectrum is partially reduced by spectral absorption of gas molecules in the earth atmosphere (like H_2O, N_2, O_2, CO_2, Ar and others) and is shown by the blue line. Moreover, the blackbody radiation at 24 °C in the room (peak at $\lambda = 10$ μm) is also presented, scaled-up by 55 times, because it would not be visible if shown in the same scale as that one for the sun.

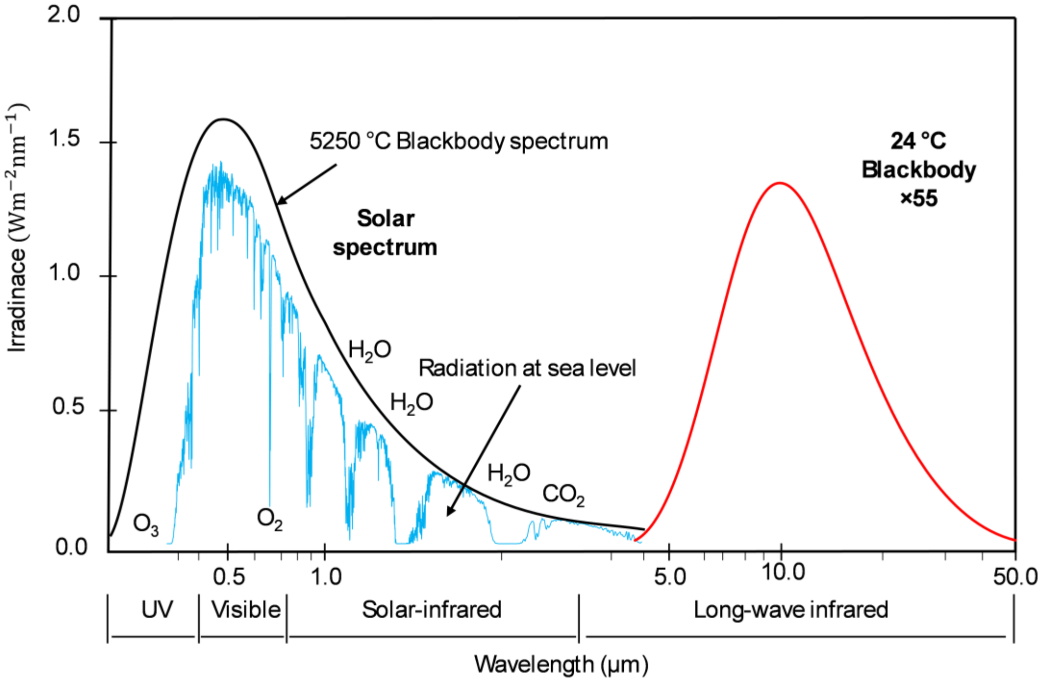

Figure S1. Illustration of solar blackbody spectrum (black curve), solar radiation at sea level (blue curve), and blackbody radiation (red curve) at 24 °C inside a room (scaled-up by 55 times for better visibility) [42].

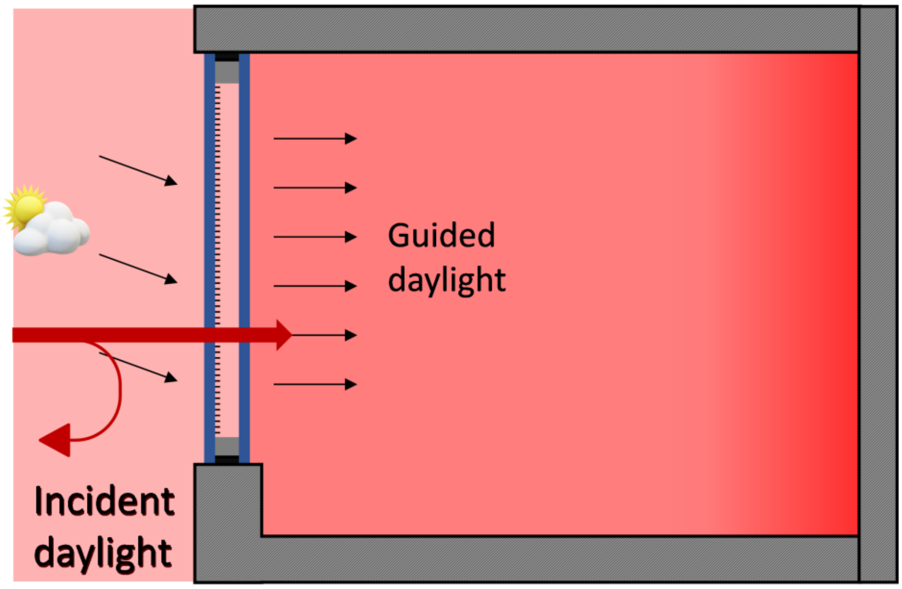

Figure S2. Schematic demonstration of the solar infrared absorption by the wall inside a room in a cold winter day without any user present. The intensity of red color symbolizes the amount of absorbed and re-emitted heat energy.

The visible and near infrared radiation (solar infrared) is absorbed inside the room. Inside the central wall this absorbed energy is converted into heat revealing a spectral black body radiation with a peak wavelength of about 10 µm for 24 °C wall temperature. Subsequently, the central wall is acting as a radiation heater, having a bit higher temperature than the rest of the room (Figure S2). Therefore, we are harvesting energy from the visible, near infrared (and mid infrared radiation) of the sun spectrum.
